# UAV Imagery Real-Time Semantic Segmentation with Global–Local Information Attention

**DOI:** 10.3390/s25061786

**Published:** 2025-03-13

**Authors:** Zikang Zhang, Gongquan Li

**Affiliations:** School of Geosciences, Yangtze University, Wuhan 430100, China; 2022710475@yangtzeu.edu.cn

**Keywords:** real-time semantic segmentation, drone imagery, feature fusion, global context information

## Abstract

In real-time semantic segmentation for drone imagery, current lightweight algorithms suffer from the lack of integration of global and local information in the image, leading to missed detections and misclassifications in the classification categories. This paper proposes a method for the real-time semantic segmentation of drones that integrates multi-scale global context information. The principle utilizes a UNet structure, with the encoder employing a Resnet18 network to extract features. The decoder incorporates a global–local attention module, where the global branch compresses and extracts global information in both vertical and horizontal directions, and the local branch extracts local information through convolution, thereby enhancing the fusion of global and local information in the image. In the segmentation head, a shallow-feature fusion module is used to multi-scale integrate the various features extracted by the encoder, thereby strengthening the spatial information in the shallow features. The model was tested on the UAvid and UDD6 datasets, achieving accuracies of 68% mIoU (mean Intersection over Union) and 67% mIoU on the two datasets, respectively, 10% and 21.2% higher than the baseline model UNet. The real-time performance of the model reached 72.4 frames/s, which is 54.4 frames/s higher than the baseline model UNet. The experimental results demonstrate that the proposed model balances accuracy and real-time performance well.

## 1. Introduction

**Figure 1:**
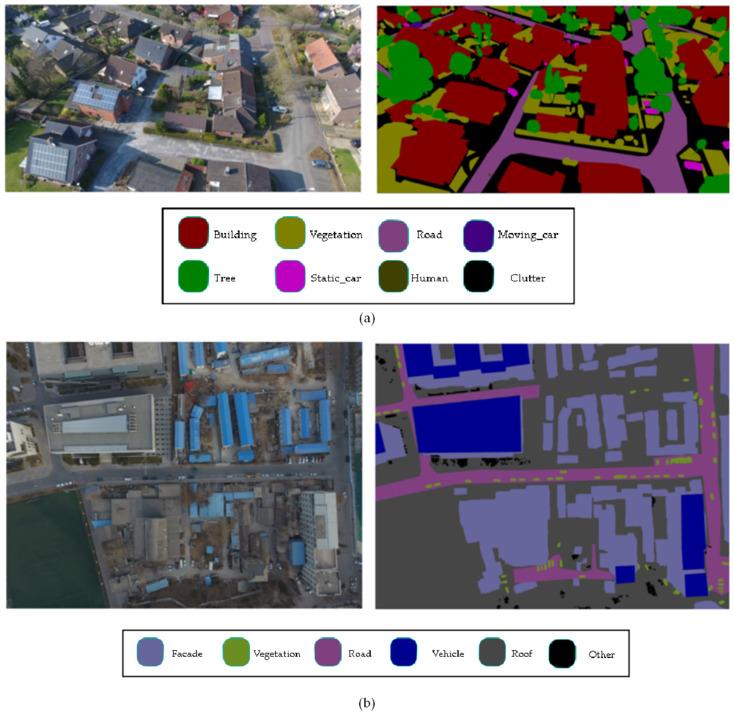
Two drone images and their semantic annotations from the UAvid and the UDD6 dataset, respectively. (**a**) Oblique projection, (**b**) Orthophoto projection.

With the continuous advancement of drone technology, low-altitude drones can collect real-time images at any time and even capture data from different angles. As shown in Figure 1, urban images collected by UAVs have high resolution and contain rich spatial information, which has great potential in disaster assessment [1,2], emergency response [3], traffic analysis [4,5], environmental monitoring [6,7], and other applications.

Semantic segmentation is a novel and challenging task in various image processing tasks for drone remote sensing. Semantic segmentation aims to classify every pixel in an image based on its semantic information. In recent years, the introduction and continuous improvement of convolutional neural networks (CNNs) have achieved remarkable results in semantic segmentation tasks [8,9].

Current drone image analysis systems rely on images captured by drones and then uploaded to a computer platform for analysis. However, in the face of urban scene problems, such as urban land-use analysis and traffic management, it is necessary to segment the categories of ground objects in the urban scene in real-time for analysis. Therefore, a lightweight semantic segmentation model is needed to segment UAV images in application. At present, many semantic segmentation methods try to increase the network depth and the number of channels of the model to capture richer semantic and spatial information so as to obtain better segmentation results. However, increasing the depth and number of channels also increases the number of parameters in the network, thus requiring more computing power from the hardware, in a way that undoubtedly increases the inference time of the model.

Pre-trained models are usually trained on massive amounts of data, which improves the efficiency and stability of training. However, for real-time semantic segmentation tasks, adopting a large pre-trained model not only improves accuracy but also brings high computational complexity. Therefore, this paper uses a lightweight pre-trained ResNet18 model as the encoder in the UNet model for feature extraction, which solves the limitations of unmanned aerial vehicle devices. This article designs a lightweight global–local attention block for decoders that require lightweight operations. This block consists of a global attention branch and a convolutional local branch, used to capture global and local contextual information about features. Finally, multi-scale methods are applied at the end of the decoder to fuse the features extracted by the encoder at different stages in order to enrich the spatial information of shallow features. Then, the fused features are added to the features of the global–local attention block, and the segmentation results are obtained through 1 × 1 convolution and upsampling.

## 2. Related Works

### 2.1. Network Architecture

In recent years, with the rapid advancement of deep learning technology, deep learning networks for real-time semantic segmentation tasks have mainly been categorized into single-branch, dual-branch, and U-shaped networks.

Single-branch networks focus primarily on the encoder, simplifying the decoder layers. The decoder is often designed to be very lightweight, reducing the parameters and computational load of the decoder and other branches, thus improving the computational speed of the model. Liu et al. [10] proposed a feature pyramid encoding block that encodes multi-scale contextual features using deep dilated convolutions at all encoder stages. On the NVIDIA TiTanV, they tested the Cityscapes and CamVid datasets at a resolution of 1024 × 512, achieving a real-time performance of 102 frames per second (fps) with accuracies of 68% mIoU and 65.4% mIoU, respectively.

Compared to single-branch networks, dual-branch networks use one branch to extract spatial information and another to model semantic contextual information. This structure balances segmentation accuracy and inference speed. Yu et al. first proposed the bilateral segmentation network, BiSeNet [11]. The spatial path branch extracts shallow spatial information to generate high-resolution features, while the context path extracts deep, rich semantic information. A feature fusion module is used to merge the two types of features. On TitanXP, testing the Cityscapes dataset at a resolution of 2048 × 1024 achieved a real-time performance of 65.5 fps with an accuracy of 74.7% more. Fan et al. proposed a short-term dense cascade network (SDCN), which adjusts the depth and number of convolution kernels to capture receptive fields of different features [12]. The spatial path branch uses only the features from stage 3, and DetailHead is employed only during training to extract more spatial detail. Testing on 1080 Ti with the Cityscapes dataset at 512 × 1024 resolution achieved 250.4 fps with an accuracy of 71.9% more.

U-shaped networks adopt an encoder–decoder structure, allowing them to sample and fuse features across different scales, extracting rich spatial and contextual information. Zhang et al. introduced a scale-aware semantic extractor that extracts semantic information at different scales, capturing the entire image’s receptive fields and semantic information [13]. Testing on Snapdragon 865 with the ADE20K dataset at 512 × 512 resolution achieved 13 fps with an accuracy of 36.1% more. Wang et al. improved model precision and accuracy by using a lightweight pre-trained model, ResNet18, as the encoder and proposed a global–local transformer block in the decoder to capture global and local context at multiple scales [14]. They also introduced a feature refinement head to fuse features from the encoder and decoder, enhancing both semantic and spatial information. Testing on the UAVid and LoveDA datasets with a 3090 GPU at 1024 × 1024 resolution achieved 115.6 fps with accuracies of 67.8% mIoU and 52.4% mIoU, respectively.

Compared to single-branch and dual-branch networks, U-shaped networks extract features at the encoder side and apply skip connections to pass these features to the corresponding decoder layers. The features from each decoder layer are fused with the up-sampled features from the encoder, serving as input for the next up-sampling layer. This fusion of multi-scale features from the encoder improves segmentation accuracy. However, U-shaped network decoders often need to perform global context feature extraction, and the complex fusion operations add extra computational burdens to the decoder. Therefore, the decoder needs lightweight fusion operations and efficient global context extraction mechanisms.

### 2.2. Global Contextual Information Module

Since CNN convolutions can only utilize local information to understand images, many recent works have attempted to construct global context modules to capture global contextual information. A popular approach is to integrate attention mechanisms into the network modules.

Hu et al. first proposed the channel attention mechanism in SENet, where spatial information is compressed into a global vector through pooling operations [15]. This global vector is then transformed non-linearly, and weights are generated using the Sigmoid function to multiply with the input. This process captures the inter-channel information in the feature map, enhancing valuable features in the channels while suppressing less useful ones. Later, Woo et al. combined channel attention with spatial attention mechanisms in sequence, capturing both spatial positional information and inter-channel information [16]. Misra et al. established interactions between different dimensions by rotating feature maps and applying pooling operations [17].

Although the above models combine channel and spatial attention mechanisms, performing 2D global pooling leads to the loss of positional information, which is crucial for generating spatial attention maps. This paper proposes a UNet-based model where the ResNet18 is used as the encoder to extract features, and a coordinate attention mechanism [18] is introduced into the decoder. A dual-branch module is designed to extract global and local contextual information, with the global branch capturing global information and the local branch focusing on local information.

## 3. Principles and Methods

### 3.1. Network Principles

**Figure 2:**
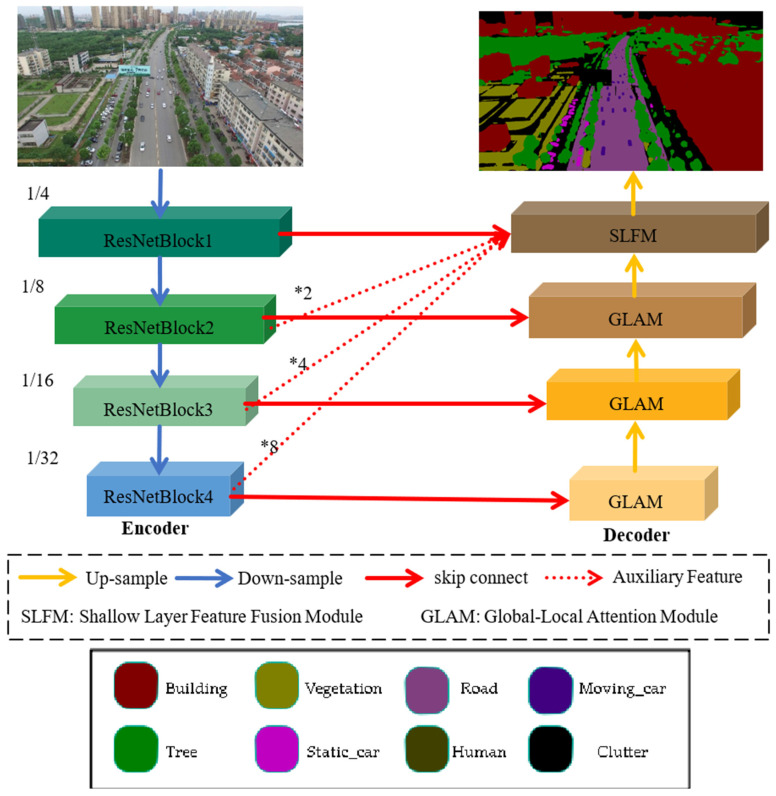
Overall structure diagram of the proposed model. (*: Upsampling).

This model is constructed based on the UNet structure, with the ResNet18 model used as the encoder and the global local attention module used as the decoder, as shown in Figure 2. The encoder of this model consists of four ResNet blocks, where the feature maps are down-sampled at each stage with a scaling factor of 2. The decoder comprises three global–local attention mechanisms and a shallow-feature fusion module: skip the connection and use the most straightforward connection operation. The global–local attention mechanism integrates corresponding feature information from the encoder to extract semantic information about the features. In contrast, the shallow-feature fusion module combines features from different scales of the encoder to enhance the spatial information of shallow features.

### 3.2. Global–Local Attention Module

**Figure 3:**
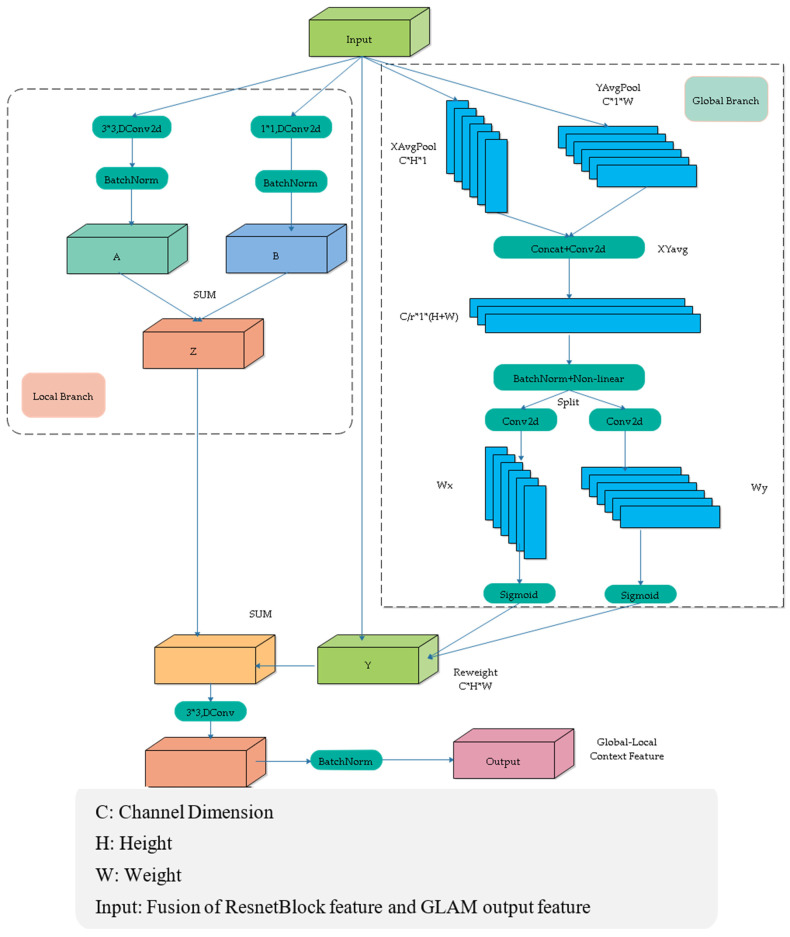
Global–local attention module network structure (GLAM).

In the global–local attention module, both a global branch and a local branch were designed to improve the model’s ability to extract and fuse contextual information from feature maps. In the global branch, pooling operations are applied along the *x*-axis and *y*-axis, compressing the information in these directions and embedding it into the feature map. However, compressing only along the *x*-axis and *y*-axis overlooks the relationships between neighboring pixels. Therefore, an additional local branch is designed, utilizing convolution operations with kernel sizes of 1 × 1 and 3 × 3 to capture the relevant information between neighboring pixels. The network structure is shown in Figure 3.

This module consists of a global attention branch and a local branch. The global attention branch utilizes coordinate attention to extract global information, considering both inter-channel relationships and positional information. The local branch applies two convolutions with kernel sizes of 3 and 1 to capture local information from the feature map. The global and local branches are then fused, enabling interaction between global and local information, which enhances the algorithm’s accuracy.

Firstly, this article inputs the feature maps of four ResNet blocks into the corresponding global–local attention modules through skip connections. The output of the deep global–local attention modules will be fused with the skip-connected feature maps as the feature input of the upper-level attention modules.

In the global branch, average pooling is performed along the horizontal and vertical directions, resulting in two 1D vectors (H × 1 and 1 × W).

(1) $$X_{avg}=XAvgPool\left(Input\right)$$

(2) $$Y_{avg}=YAvgPool\left(Input\right)$$

These vectors are concatenated in the spatial dimension, followed by a 1 × 1 convolution to reduce the channel dimensions.

(3) $${XY}_{avg}=Conv2d(Concat\left(X_{avg},\;Y_{avg}\right))$$

Then batch normalization (BN) and a non-linear activation are applied to encode the spatial information in the vertical and horizontal directions. The result is then split back into horizontal and vertical components, and a 1 × 1 convolution is used to restore the channel count.

(4) $$F_{x},\;F_{y}=Split\left(Relu\left(BatchNorm\left({XY}_{avg}\right)\right)\right)$$

(5) $$W_{x},\;W_{y}=\delta \left(Conv1\times 1\left(F_{x}\right)\right),\delta \left(Conv1\times 1\left(F_{y}\right)\right)$$

Finally, a gating mechanism generates the weights, and the global contextual features are obtained through weighted summation.

(6) $$Y=X⊙W_{x}⊙W_{y}$$

In the formula, *Conv*1 × 1 represents a convolution operation with a kernel size of 1 × 1; $X_{avg}$ and $Y_{avg}$ represent the coordinate information embedding in the horizontal and vertical directions, respectively; δ represents the Sigmoid function; $W_{x}$, $W_{y}$ represent the weights in the horizontal and vertical directions; ⊙ represents element-wise multiplication.

For the local branch, The Input∈R^(C × H × W) is passed through convolution operations with kernel sizes of 3 × 3 and 1 × 1, followed by a BN (Batch Normalization) layer. The output of the local branch *Z* is obtained by element-wise addition of the processed features. This process can be described as follows:

(7) $$A=BatchNorm\left(Conv3\times 3\left(Input\right)\right)$$

(8) $$B=BatchNorm\left(Conv1\times 1\left(Input\right)\right)$$

(9) Z=A⊕B

In the formula, ⊕ represents the element-wise addition operation. We add the features from the global and local branches. Then, we apply a depth-wise separable convolution with a kernel size of 3 × 3, followed by a BN (Batch Normalization) layer to obtain the final fused features. This process can be described as follows:

(10) $$Output=BN\left(DWConv3\times 3\left(Y⊕Z\right)\right)$$

In the formula, *DWConv*3 × 3 represents a depth-wise separable convolution with a kernel size of 3 × 3.

### 3.3. Shallow Layer Feature Fusion Module

**Figure 4:**
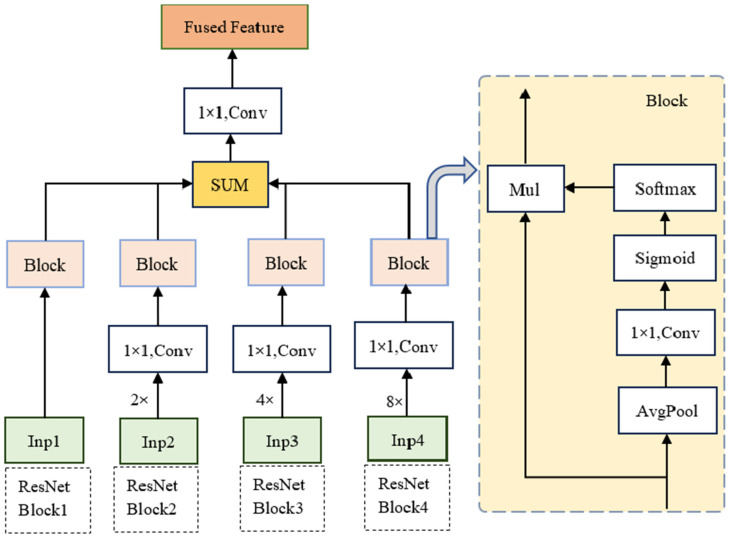
Shallow layer feature fusion module network structure (SLFM).

We introduce a shallow-feature fusion module to enhance the spatial details of shallow layer features and improve classification accuracy, as shown in Figure 4. First, the features from the other three ResNet blocks undergo up-sampling and convolution operations to align their size and dimensionality with those of the features from the first ResNet block. Subsequently, global feature representation is obtained through a global average pooling operation in the spatial dimension. A 1 × 1 convolution is then applied to model the inter-channel relationships, and a Sigmoid activation function is used to generate channel weights.

Next, the global features from the four scales are concatenated along the second dimension to obtain the corresponding output X. The weights for each scale are then derived using the softmax function along the second dimension. Finally, the generated weights are multiplied element-wise with the corresponding inputs and summed together.

## 4. Experimental Process and Analysis

In this paper, the model is trained on two datasets of UAV remote sensing imagery, UAVid [19], UDD6 [20], and LoveDA [21]. The model’s accuracy and inference speed are tested and compared with several state-of-the-art models proposed in recent years.

### 4.1. Dataset

The UAVid dataset is a high-resolution dataset (3840 × 2160 and 4096 × 2160) captured by drones, designed explicitly for urban scene semantic segmentation tasks. It contains 42 sequences. Each sequence includes ten images, resulting in a total of 420 images. The dataset is divided into 20 training sequences, 7 validation sequences, and 15 test sequences; that is, the training set has 270 images, and the validation set has 150 images, which are independent of each other. The UAVid dataset includes eight types of ground objects: buildings, roads, static vehicles, trees, low vegetation, people, moving vehicles, and cluttered backgrounds.

UDD6 is a dataset for the understanding and reconstruction of aerial scenes collected and labeled by the Graphics and Interaction Laboratory of Peking University using DJI Phantom 4 drones at a height between 60–100 m in four cities in China. The resolution of the data is 4 k (4096 × 2160) or 12 M (4000 × 3000) and includes a variety of urban scenes. The dataset is divided into 85 training images, 35 verification images and 20 test images, and the training images and test images are independent of each other. This dataset is divided into facades, roads, vegetation, cars, roofs, and others.

The LoveDa dataset constructs an urban–rural domain adaptive land-cover dataset, which includes 5987 high-resolution images of 0.3 m from three different cities, Nanjing, Changzhou, and Wuhan. Each image has a resolution of 1024 × 1024 and includes seven categories: background, buildings, roads, water bodies, bare soil, forest land, and cultivated land. According to the official LoveDA data, the training set consists of 2521 images, the validation set consists of 1670 images, and the test set consists of 1796 images.

### 4.2. Experimental Conditions

The experiments were conducted using the PyTorch 2.1.0 deep learning framework and the CUDA 11.8 library, with Python version 3.8, on a Windows 11 operating system. Model training and testing were performed on an Intel Xeon Gold 6348 CPU (Inter, Shanghai, China) and an NVIDIA GeForce RTX 3090 24 GB GPU (Nvidia, Shanghai, China) with 256 GB of RAM.

### 4.3. Performance Evaluation Metrics

In this paper, the evaluation metrics include the mean Intersection over Union (mIoU) to measure the prediction accuracy of the algorithm, and the frames per second (FPS) to assess the computational speed of the proposed model on the experimental hardware. The mIoU is calculated as the average ratio of the intersection and union between the ground truth and the predicted values for all classes. The formula for mIoU is as follows:

(11) $$mIoU=\frac{1}{m+1}\sum_{i=0}^{m}\frac{p_{ii}}{\sum_{j=0}^{m}p_{ij}+\sum_{j=0}^{m}p_{ji}-p_{ii}}$$

In the formula, m represents the number of pixel classes; $p_{ij}$ is the number of pixels whose true class is i but are predicted as class j; $p_{ii}$ is the number of pixels correctly predicted as a class, i, where the actual class is also i.

FPS is calculated as the ratio of the number of frames N to the time required to process them, representing the model’s computational speed:

(12) $$f_{FPS}=\frac{N}{\sum_{i=1}^{N}T_{façade}}$$

In the formula, N represents the number of frames, and $T_{i}$ is the time required to process the *i*-th image.

F1 is a statistical indicator used to measure the accuracy of binary classification models. It is the harmonic mean of precision and recall, providing a single metric to evaluate the overall performance of the model. The range of F1 values is from 0 to 1, with higher values indicating better performance of the model.

(13) $$F1=\frac{2*TP}{TP+FP+TP+FN}$$

Among them, TP correctly predicted the positive class; that is, it was originally a positive class and recognized the positive class as well; TN correctly predicted the negative class; that is, it was originally a negative class and recognized a negative class as well; FP is incorrectly predicted as a positive class, meaning it was originally a negative class but recognized as a positive class; FN was incorrectly predicted as a negative class, meaning it was originally a positive class but was recognized as a negative class.

### 4.4. Comparative Analysis of Experimental Results

To validate the impact of each module in this paper on mIoU and FPS, we conducted evaluations on the UDD6 dataset and the UAVid dataset. The evaluation metrics, mIoU and FPS, were used to analyze the impact of the modules on the model’s segmentation accuracy and inference speed.

During training, the AdamW optimizer was used to train the models, with the base learning rate set to 0.0006. A cosine annealing strategy was applied to adjust the learning rate. The batch size was set to 8, and the number of epochs to 40. The cross-entropy loss function was used as the loss function during training. In the experiments, images from both datasets were padded and cropped into eight patches of 1024 × 1024 pixels. Data augmentation techniques, such as random vertical and horizontal flips and random brightness adjustments, were applied to the input images.

#### 4.4.1. UDD6 Ablation Study

In this paper, different combinations of modules were functionally verified on the UDD6 dataset, with the results shown in Table 1. The baseline model used was Unet.

In Table 1, the mIoU, F1, and OA metrics were used to test the effect of the modules on network performance. It can be observed that the added modules contributed to a particular improvement in mIoU compared to the baseline model. After integrating all proposed modules, the mIoU of this model reached 67%, an improvement of 21.2% compared to the baseline model. Adding the GLAM module to the baseline model has significantly improved the recognition of various types, especially in the Façade class, Road class, Vegetation class, and Roof class, where the improvement is better, with an increase of 18%, 13.6%, 30.5%, and 27.9%, respectively. When both the GLAM and SLFM modules are added to the baseline model, compared to a single GLAM module, the segmentation performance is better in the Road class, Vegetable class, and Other class, with improvements of 4.5%, 1.3%, and 1.2%, respectively. For other categories, the effect remained the same or slightly improved, with a 1.4% increase in mIoU, a 0.7% increase in the F1 score, and a 0.4% increase in OA.

**Figure 5:**
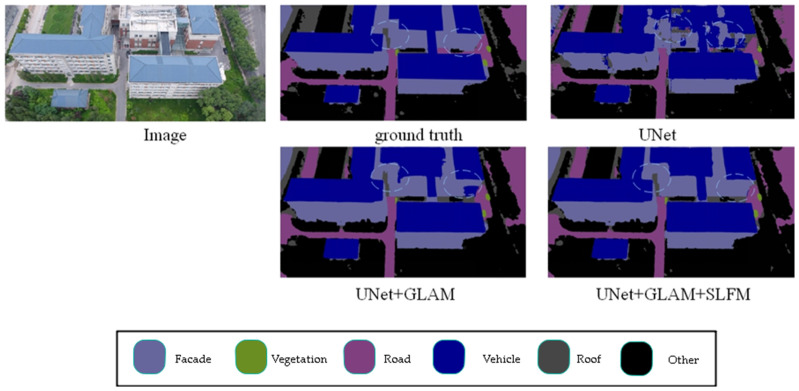
UDD ablation study. (The image name is: DJI_0538.JPG).

Figure 5 presents the results of ablation experiments on the UDD6 dataset, comparing different scenarios. The highlighted sections indicate the differences between various modules. Adding the modules enhances the model’s ability to segment details and small objects more accurately. For example, when comparing the segmentation results from the first and second rows, adding the modules improves the accuracy and enables precise segmentation of object edges. The experimental results demonstrate the effectiveness of the proposed modules.

#### 4.4.2. UAVid Ablation Study

In this paper, the functionality of different module combinations was verified on the UAVid dataset, with results shown in Table 1, where the baseline refers to Unet.

The results in Table 2 show that the modules remain effective on the UAVid dataset. The addition of these modules to the baseline brought about an increase in the segmentation accuracy, measured by mIoU (mean Intersection over Union). Specifically, adding individual and combined modules resulted in 9% and 10% improvements in mIoU, respectively, compared to the baseline.

**Figure 6:**
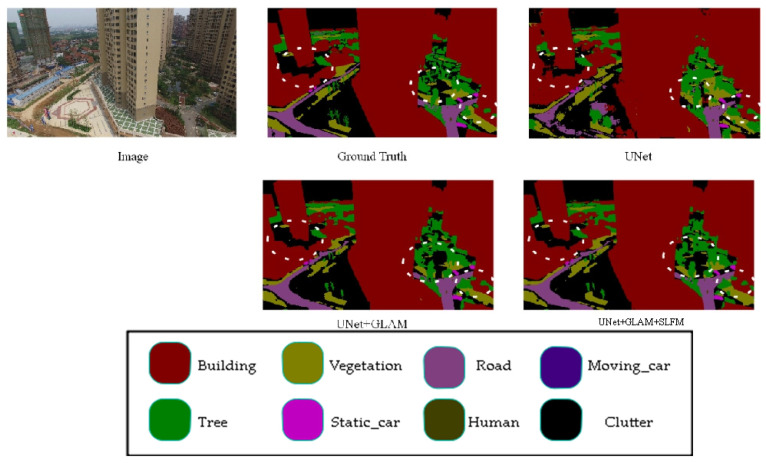
UAVid ablation study. (The image name is: Seg5\000400.png).

Figure 6 showcases the results of ablation experiments on the UAVid dataset for the proposed model. The results show that the proposed model enhances the detection of small objects and details. Adding the GLAM module to the baseline model has improved the precise segmentation of object edge details. Building on this, incorporating the SLFM module has enhanced the segmentation of small targets, such as vegetation recognition, making it closer to accurate labels. These results demonstrate the generalization capability of the proposed modules, effectively improving the model’s segmentation accuracy while achieving a balanced trade-off between precision and speed.

#### 4.4.3. UAVid Comparison Experiment

To verify the proposed algorithm, we conducted a comparative study on the UAVid dataset with various network models, such as CGNet [22], ContextNet [23], DABNet [24], EDANet [25], FPENet [10], LinkNet [26], SegNet [8], LCNet [27], and UnetFormer [14]. The comparison results are shown in Table 3. The proposed algorithm achieved the highest mIoU of 68%. The proposed model outperformed CGNet, ContextNet, DABNet, EDANet, FPENet, LinkNet, and SegNet in UAVid class recognition, and achieved comparable performance with UnetFormer. Specifically, our model outperforms UnetFormer by 3% in static vehicle recognition.

**Figure 7:**
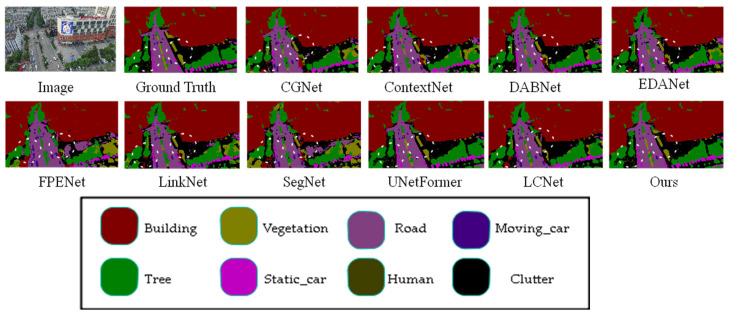
Comparison of Visual Results from Different Models on UAVid Dataset. (The image name is: seq1\00800.png).

This article compares the prediction results of different models on the Uavid dataset in Figure 7, with some apparent differences marked with white wireframes. From the figure, it can be observed that the proposed model demonstrates superior segmentation accuracy for edge details compared to other models. For instance, in the areas of the house and the grass beside the road highlighted in the white box, the proposed model achieves more precise recognition of the house edges and the grass along the road than the other models.

#### 4.4.4. UDD6 Comparison Experiment

To verify the robustness of the model, we further conducted comparative experiments on the UDD6 dataset, and the results are shown in Table 4. The table shows that the proposed model achieved a mIoU of 67%. The proposed model outperformed other models in recognizing all categories on the UDD6 dataset. Specifically, it surpassed the highest performance of different models in the road category by 0.7%, in the vegetation category by 2%, in the vehicle category by 1%, and in the roof category by 1.5%.

The data indicates that the proposed model significantly improves recognition accuracy for the façade, road, vegetation, and roof categories compared to other models. The data suggests that the proposed model is more precise in segmenting the contours of more prominent categories.

**Figure 8:**
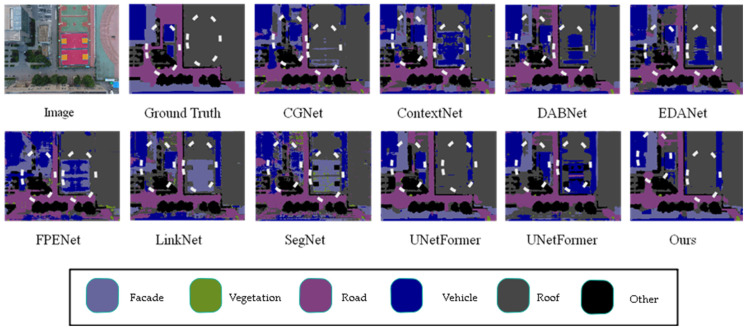
Comparison of visual results from different models on the UDD6 dataset. (The image name is: DJI_0667.JPG).

This article visually compares the prediction results of different models on the UDD6 dataset in Figure 8, with significant differences highlighted by white bounding boxes. From the figure, it can be observed that the proposed model demonstrates greater accuracy in segmenting edge details compared to other models. For instance, the segmentation of roofs and walls is more precise, with contours that closely match the ground truth labels. Additionally, the proposed model correctly identifies instances where other networks misclassify labels.

#### 4.4.5. LoveDA Ablation Study

In order to verify the universality of the model in this article, the LoveDa dataset was selected, which includes urban and rural scenarios to validate the universality of this article. The results are shown in the Table 5. The GLAM module proposed in this article has significantly improved in various categories on the baseline model, with improvements of 8.4%, 17.8%, and 9.6% in the road, bare soil, and cultivated land categories, 8.6% in mIoU, 6.7% in the F1 value, and 5.5% in the OA value. After adding the SLFM module, the model in this article showed good improvement in recognition rates for various types, with improvements of 2.8%, 1.9%, and 2.9% in the background, building, and bare soil categories, respectively. It also showed a 2% increase in mIoU, a 1.5% increase in the F1 value, and a 1.5% increase in the OA value.

**Figure 9:**
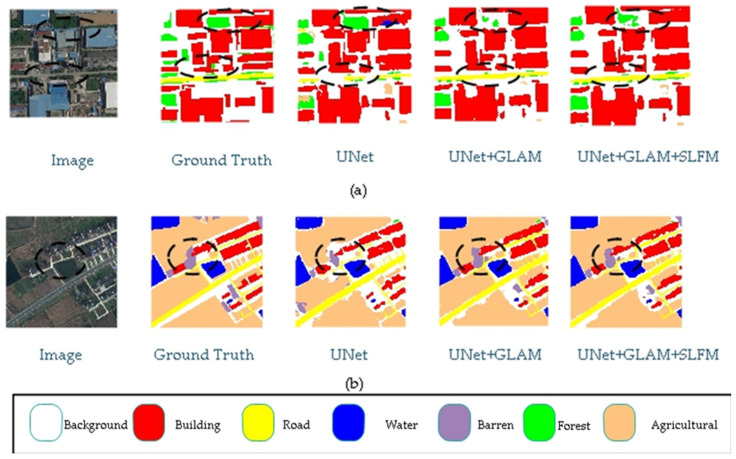
LoveDA ablation study. (The (**a**) image name is: urban\4178.png, the (**b**) image name is: rural\2522.png).

This article visualizes the ablation experiments conducted on the LoveDA dataset. As shown in Figure 9, the model proposed in this paper performs better in edge segmentation compared to the baseline model, and performs better in edge segmentation between adjacent different objects. In Figure 9a, the black wireframe shows better segmentation performance for the edges of buildings and roads compared to the baseline model.

#### 4.4.6. LoveDA Comparison Experiment

This article conducted comparative experiments with other models on the LoveDA dataset, as shown in Table 6. The model proposed in this article has shown certain improvements in various types of recognition compared to other models, achieving the highest recognition rate in each type. Among them, compared with other models with the highest recognition rates, the background class improved by 0.6%, the building class improved by 1%, the road class improved by 1.3%, the water body class improved by 0.6%, the bare soil class improved by 0.7%, the forest class improved by 0.9%, and the mIoU class improved by 1.3%, The F1 value increased by 1%, and the OA value increased by 0.8%. From a data perspective, the model proposed in this article has improved its ability to recognize regular edges for similar buildings and roads.

**Figure 10:**
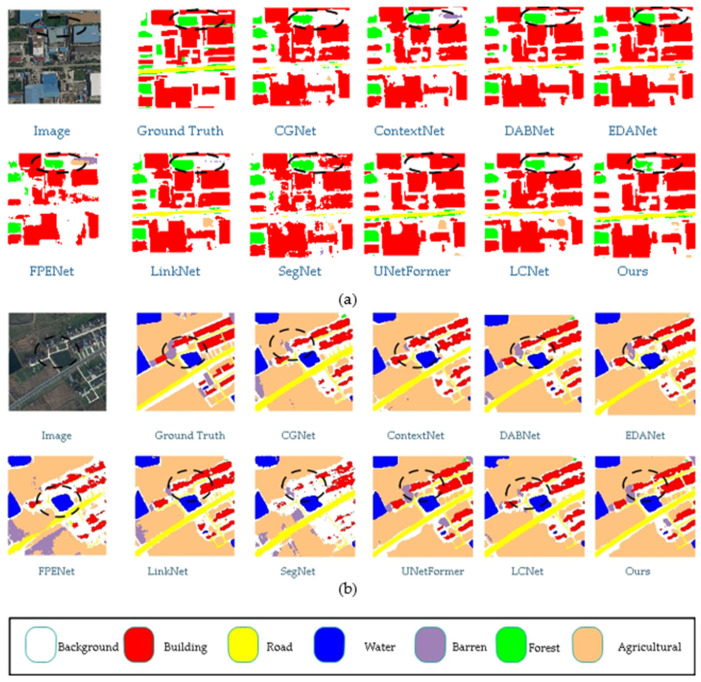
Comparison of visual results from different models on the LoveDA dataset. (The (**a**) image name is: urban\4178.png, the (**b**) image name is: rural\2522.png).

This article compares the visualization results of each model, as shown in the figure. In Figure 10a, compared with other models, the model proposed in this paper has significantly improved the object edge segmentation effect, especially in the black wireframe where effective segmentation was performed on the edge parts contained in adjacent objects. In Figure 10b, the model proposed in this article performs well in segmenting rural scenes. For example, in the black wireframe, the segmentation effect of bare soil and forest edges is closer to the real segmentation.

#### 4.4.7. Comparison of Computational Complexity

This article will present a comparison between the parameter count, computational complexity, and inference speed of different models, as shown in Table 7. The model proposed in this article has a slight advantage in terms of speed. In terms of inference speed, the FPS of our model is 72.4, slightly higher than CGNet, FPENet, and FPENet. However, the model in this article has a parameter count of 12.1 M, which is only lower than SegNet and slightly lower than LinkNet and UnetFomer. In terms of computational complexity, it is 49.24 G, only lower than LinkNet and SegNet, and slightly lower than UnetFomer. Based on the segmentation performance of Table 4, Table 5 and Table 6 on various datasets, it can be seen that our model has effectively improved segmentation accuracy compared to other models. This advantage makes our model suitable for the real-time segmentation of low- altitude drone images.

## 5. Discussion

The article designs a global–local attention module for extracting global features and a shallow-feature fusion module. The global–local attention module adopts a dual-branch structure, where a lightweight attention mechanism is introduced into the global branch to account for the real-time requirements of drone tasks. The global features extracted from a single global branch lack certain local information. Therefore, an additional local branch is designed to capture local information and then fused with global features to address this issue. As shown in Figure 5, the visualization results of the global–local module added to the baseline model enhance the recognition of walls, making it more aligned with the actual labels.

The low-level-feature fusion module utilizes features from the U-Net’s down-sampling layers. The shallowest down-sampled features contain rich spatial information but have limited semantic information, while the deeper ones are rich in semantic information but lack spatial details. This article designs a shallow-feature module to fuse deep and shallow features, which are then output for the decoder’s feature fusion, solving the problem of deep feature output from the decoder lacking spatial information, especially for small object recognition, as shown in Figure 6. Although this module is added to the skip connections in the U-Net to reduce its impact on model speed, it is only added at the top layer, thus not significantly affecting the overall speed of the model, as shown in Table 3.

However, the recognition performance of the model proposed in this article is not satisfactory in some classes. As shown in Table 2 and Table 3, the recognition performance of our model in the Human class on the UAVid dataset is poor. Combined with the performance of our model on the UDD6, UAVid, and LoveDA datasets, as shown in Figure 5, Figure 6, Figure 7, Figure 8, Figure 9 and Figure 10, our model performs well in object edge segmentation. However, there is still room for improvement in small object segmentation.

## 6. Conclusions

This paper proposes a real-time semantic segmentation algorithm based on a channel attention mechanism. The model primarily comprises an encoder, a global–local attention module, and a shallow-feature fusion module.

First, a multi-scale shallow-feature fusion module is designed in the top-level skip connections to enhance features by integrating the characteristics from different stages of the encoder.

Second, the proposed global–local attention module effectively combines global and local information in channel and spatial dimensions, resulting in better feature extraction.

The proposed model achieves a real-time performance of 72.4 FPS with an input resolution of 1024 × 1024 on the UAVid test set, with an accuracy of 68% mIoU. Similarly, the UDD6 test set achieves an accuracy of 67% mIoU at the exact resolution, demonstrating the effectiveness of the proposed model. The proposed model achieves a better balance between accuracy and real-time performance than other algorithms.

## Figures and Tables

**Table 1:** Effect of modules on the UDD6 dataset.

Model	Per-Class IoU (%)						mIoU (%)	F1 (%)	OA (%)
	Façade	Road	Vegetation	Vehicle	Roof	Other			
Baseline	44.1	51.8	30.2	65.6	37.3	75.0	45.8	61.9	71.2
Baseline + GLAM	62.1	65.4	60.7	77.3	65.2	88.7	65.8	79.5	84.4
Baseline + GLAM + SLFM	62.1	69.9	62.0	77.4	66.0	89.9	67.2	80.2	84.8

**Table 2:** Effect of the modules on the UAVid dataset. (Online Test).

Model	Per-Class IoU (%)								mIoU%
	Clutter	Building	Road	Tree	Vegetation	Moving Car	Static Car	Human	
Baseline	57	79	74	75	57	66	38	25	58
Baseline + GLAM	68	86	80	80	63	74	57	31	67
Baseline + GLAM + SLFM	68	87	81	80	63	74	59	31	68

**Table 3:** The effectiveness of different algorithms on the UAVid dataset (Resolution: 1024 × 1024).

Model	Clutter	Building	Road	Tree	Vegetation	Moving Car	Static Car	Human
CGNet	53	75	74	71	54	62	32	20
ContextNet	54	76	73	72	53	61	31	18
DABNet	57	79	76	75	58	67	38	25
EDANet	57	79	76	74	56	65	38	24
FPENet	47	69	67	65	46	44	17	0
LinkNet	54	78	73	74	55	66	36	13
SegNet	50	74	71	68	47	60	23	18
UnetFormer	68	87	81	80	63	74	56	31
LCNet	55	76	76	73	55	62	30	10
Ours	68	87	81	80	63	74	59	31

**Table 4:** Comparison of different algorithms on UDD6 dataset (Resolution: 1024 × 1024).

Model	Per-Class IoU (%)					mIoU	F1	OA
	Façade	Road	Vegetation	Vehicle	Roof			
CGNet	44	57.8	33.1	60.6	43.3	47.8	64.0	72.7
ContextNet	44.3	58.9	31.7	67.8	52.4	51	66.6	76.8
DABNet	47.4	62.1	42.3	59.8	53.2	53	68.9	76.5
EDANet	44.6	57.8	36.6	62.2	48	49.8	66.0	74.7
FPENet	41.3	47	20.5	60	36.7	41.1	57.0	68.7
LinkNet	43.8	56.2	20.2	65.2	44.4	46	61.4	73.3
SegNet	32.6	46.2	20.9	57.3	31.7	37.8	53.6	64.9
UnetFormer	62.8	69.2	60	75	64.5	66.4	70.5	60.1
LCNet	39.9	47.3	18.7	56.9	39.8	40.5	56.4	68.6
Ours	62	69.9	62	76	66	67.2	80.2	84.8

**Table 5:** Comparison of different algorithms on LoveDA dataset (Resolution: 1024 × 1024).

Model	Per-Class IoU (%)							mIoU (%)	F1 (%)	OA (%)
	Background	Building	Road	Water	Barren	Forest	Agriculture			
Unet	58.4	64.3	59.9	73.4	43.2	49.8	65.6	59.2	73.9	75.8
Unet + GLAM	63.5	69.8	68.3	77.9	61.0	56.7	75.2	67.8	80.6	81.3
Unet + GLAM + SLFM	66.3	71.7	69.7	79.4	63.9	57.9	76.7	69.8	82.1	82.8

**Table 6:** Effect of the modules on the LoveDA dataset.

Model	Per-Class IoU (%)							mIoU (%)	F1 (%)	OA (%)
	Background	Building	Road	Water	Barren	Forest	Agriculture			
CGNet	56.8	59.1	55.1	71.6	36.6	47.7	63.8	55.9	71.1	74.1
ContextNet	56.8	57.7	55.4	72.2	37.6	47.8	64.8	56.1	71.2	74.2
DABNet	59.0	64.5	61.8	74.5	49.1	51.3	68.4	61.2	75.6	77.1
EDANet	58.1	63.2	60.1	73.7	46.2	50.0	67.3	59.8	74.4	76.2
FPENet	52.8	45.8	37.9	64.6	25.0	41.1	56.0	46.2	62.2	68.0
LinkNet	58.3	63.0	59.8	73.8	43.5	50.1	67.3	59.3	74.1	76.2
SegNet	53.4	51.8	46.9	60.4	28.0	46.3	58.3	49.3	65.4	69.3
UNetFormer	65.7	70.7	68.5	78.8	63.2	57.0	75.6	68.5	81.1	82.0
LCNet	57.1	61.9	57.0	73.0	43.0	48.3	66.0	58.0	73.0	75.2
Ours	66.3	71.7	69.7	79.4	63.9	57.9	76.7	69.8	82.1	82.8

**Table 7:** Comparison of parameter quantity and speed among different models.

Model	Params	Flops	Speed
CGNet	492 K	14.24 G	64.7
ContextNet	874 K	3.59 G	147.7
DABNet	753 K	21.6 G	81.3
EDANet	683 K	17.83 G	96.3
FPENet	114 K	3.15 G	71.7
LinkNet	11.5 M	49.0 G	113.6
SegNet	29.4 M	645.12 G	18.5
UNetFormer	11.8 M	46.97 G	83.1
LCNet	508 K	15.98 G	102.1
Ours	12.1 M	48.24 G	72.4

## Data Availability

All data included in this study are available upon request by contact with the corresponding author.

## Abbreviations

The following abbreviations are used in this manuscript.

mIoU	mean Intersection over Union
GLAM	global–local attention module
SLFM	shallow layer feature fusion Module
